# Functional and Neurological Outcomes After Spine Surgery and Neurorehabilitation for Chronic Discogenic Low Back Pain: A Prospective Observational Pre–Post Study

**DOI:** 10.3390/healthcare14020258

**Published:** 2026-01-21

**Authors:** Monika Michalak, Adam Druszcz, Maciej Miś, Marcin Miś, Małgorzata Paprocka-Borowicz, Joanna Rosińczuk

**Affiliations:** 1 Department of Nursing and Obstetrics, Wroclaw Medical University, 51-618 Wroclaw, Poland; monika.michalak@umw.edu.pl; 2 Department of Neurosurgery, Provincial Specialist Hospital in Legnica, 59-220 Legnica, Poland; dr.druszcz@gmail.com; 3 Department of Neurosurgery, University Centre of Neurology and Neurosurgery, Wroclaw Medical University, 50-556 Wroclaw, Poland; mis.neurochirurg@gmail.com (M.M.); marcin.mis@gmail.com (M.M.); 4 Department of Neurological Rehabilitation, Regional Specialist Hospital in Wroclaw, 51-128 Wroclaw, Poland; malgorzata.paprocka-borowicz@wssk.wroc.pl

**Keywords:** low back pain, intervertebral disc degeneration, lumbar vertebrae, spine surgery, rehabilitation, neurological examination, functional outcome, pain measurement

## Abstract

**Background:** Discogenic low back pain (LBP) is a significant therapeutic and social problem. Discopathy is associated with neurological symptoms that severely disrupt the patient’s functional status. Regardless of the choice of neurosurgical procedure for discopathy, its effectiveness highly varies. **Aims:** This study aimed to assess the effectiveness of neurosurgical treatment and neurorehabilitation procedures based on a comprehensive analysis of a number of neurological symptoms and the functional status of patients with chronic discogenic LBP. **Material and Methods:** This study involved 110 patients (56 women and 54 men) who underwent first-ever lumbar spine surgery. Before the surgery and 3 months after the hospital discharge, all patients were subjected to neurological examination and comprehensive assessment of neurological symptoms. **Results:** After the procedure, improvement was shown in sensory disturbance (*p* < 0.0001), pain (*p* < 0.0001), and sexual dysfunction (*p* < 0.0001). The results of lower limb paresis, monoplegia, and sphincter complications remained non-significant. A reduction in scoliosis (*p* = 0.0040) and lumbar pain (*p* < 0.0001) was observed. There was a reduction in pain in the lower leg (*p* = 0.0136) and foot (*p* = 0.0122) during movements. Improvement in passive and active mobility as well as pain reduction in the lumbar spine area were demonstrated (*p* < 0.0001). There was significant improvement in the knee and ankle reflexes (*p* < 0.0001). There were no significant changes in the superficial sensation. In the functional assessment, an improvement in the toe-to-floor test of 13.3 cm was confirmed (*p* < 0.0001), while there was no difference in the Lasègue’s test. **Conclusions:** The general and neurological condition of patients with LBP significantly improved after the spine surgery. The improvement included mainly a reduction in pain and sensory disturbances, return of deep reflexes, and increased mobility of the lower limbs and spine.

## 1. Introduction

Chronic low back pain (LBP), defined as a feeling of pain, stiffness, or tension between the costal arches and the lower gluteal folds, affects approximately 60% of all adults at some point in their lives [1]. The annual incidence of LBP is approximately 15% (12–33%), while the point prevalence in the population is estimated at 30% (according to various sources 12–65%) [2,3]. Based on a systematic review of the literature on studies conducted in the period from 1980 to 2009 (165 studies from 54 countries, including Poland), LBP is a significant health problem worldwide, affecting mainly women and people at the age of 40–80 [2].

The latest data from 2023, prepared by the Global Burden of Disease Study 2021 Low Back Pain Collaborators [4], show that LBP affected 619 million people around the world in 2020. Additionally, the global age-standardized rate of years lived with disability (YLDs) was 832 per 100,000. Despite a slight decline in age-adjusted rates over the past three decades, it is forecasted that more than 800 million people will suffer from LBP by 2050 globally. As the population ages that problem will intensify in the coming decades, especially in Western societies; therefore, a more detailed assessment and analysis of its impact on the quality of life of individuals and the level of health of societies is extremely important [3].

Radiculopathy, spinal stenosis, and facet syndrome represent distinct but interrelated contributors to LBP, each characterized by unique pathological mechanisms [5,6]. Radiculopathy involves compression or irritation of spinal nerve roots, leading to radiating pain, tingling, and weakness along the affected nerve’s pathway [7]. In contrast, spinal stenosis refers to the narrowing of the spinal canal, often causing pressure on the spinal cord or nerve roots, resulting in pain, numbness, and difficulty walking [8]. Facet syndrome originates from inflammation or degeneration of the facet joints connecting vertebrae, causing localized pain and stiffness [9]. Understanding the specific characteristics of these conditions is crucial for accurate diagnosis and targeted interventions, ensuring that healthcare professionals can tailor treatment strategies to address the underlying mechanisms and alleviate patients’ LBP effectively [10].

Degenerative changes in the intervertebral disc are a major contributor to LBP, posing a significant therapeutic challenge, especially for young, active individuals [11]. Discopathy, characterized by symptoms like gradual lower back pain progressing to the buttocks and thigh, is prevalent, affecting a considerable percentage of individuals over 40 [12]. Conservative treatments are common for intervertebral disc degeneration (IDD), but surgical interventions are considered if conservative measures fail and the patient’s condition deteriorates, especially in cases of chronic pain. However, evaluating the efficacy of surgery remains challenging due to varied methods, patient criteria, and inconsistent pain assessments [13,14]. Surgical options include discectomy, laminectomy, microdiscectomy, stabilization, implantation, decompression, or minimally invasive surgery, tailored to each patient’s clinical context [15,16].

Regardless of the choice of neurosurgical procedure for the treatment of IDD, its effectiveness highly varies [17]. It depends on a number of factors, such as the severity of the disc damage, the location of the disc herniation, the age of the patient, the general health condition and the experience and skills of the surgeon, as well as participation in post-operative neurorehabilitation [18,19]. Neurorehabilitation plays a crucial role in the postoperative management of patients undergoing spine surgery [20]. It encompasses various strategies aimed at restoring neurological function, reducing pain, and enhancing functional mobility. Comprehensive rehabilitation programs include targeted physical therapy, exercise regimens focusing on strength and flexibility, neuromuscular re-education, and pain management techniques [21]. Properly structured neurorehabilitation following spine surgery can significantly influence patient outcomes, accelerating recovery and improving quality of life [19,22,23].

In addition to exercise-based and functional rehabilitation, contemporary neurorehabilitation programs following lumbar spine surgery frequently incorporate physical agents aimed at pain modulation, reduction in inflammation, and facilitation of tissue healing. Modalities such as low-level and high-intensity laser therapy (LLLT/HILT), magnetic field therapy, therapeutic ultrasound, shock wave therapy (ESWT), and transcutaneous electrical nerve stimulation (TENS) are commonly used as adjunctive interventions [24,25,26,27]. These physical agents have been shown to exert analgesic effects, improve local microcirculation, modulate neuromuscular activity, and support functional recovery, particularly in patients with persistent postoperative pain or sensory disturbances. Although their efficacy may vary depending on treatment parameters and patient characteristics, evidence from clinical studies and systematic reviews suggests that, when appropriately applied, physical modalities can enhance the overall effectiveness of comprehensive neurorehabilitation programs following spine surgery [20].

Outcomes following neurosurgical treatment for IDD may vary substantially between individual patients. Surgical intervention is typically reserved for cases associated with progressive neurological deficits, significant functional impairment, or failure of adequately applied conservative treatment, and the decision to operate requires careful consideration of potential risks and benefits for each patient. While neurosurgical decompression aims to relieve neural compression, postoperative recovery and resolution of neurological symptoms are influenced by multiple factors, including the effectiveness of subsequent neurorehabilitation [28]. Despite the widespread clinical use of postoperative rehabilitation following spine surgery, there remains a relative paucity of studies that comprehensively evaluate the combined effectiveness of neurosurgical treatment and structured neurorehabilitation in alleviating neurological symptoms associated with discopathy. Addressing this gap is essential to better understand early postoperative neurological recovery and to optimize integrated treatment pathways for patients with chronic discogenic LBP.

This study aimed to assess the effectiveness of neurosurgical treatment and neurorehabilitation procedures in terms of reducing or eliminating unfavorable symptoms resulting from the compression of the intervertebral disc on neural structures based on a comprehensive analysis of a number of neurological symptoms. The primary outcome was the overall improvement in neurological function, operationalized as changes in key neurological signs and symptoms associated with nerve root compression, including motor function, tendon reflexes, and sensory disturbances, assessed before surgery and at the 3-month follow-up. The secondary outcomes included changes in individual neurological symptoms (e.g., pain intensity, numbness, tingling, reflex abnormalities), functional mobility, and other patient-reported complaints potentially related to discogenic neural compression, assessed at the same time points.

## 2. Materials and Methods

### 2.1. Ethical Considerations

This prospective observational pre–post study was approved by the Bioethics Committee of the Wroclaw Medical University (no. KB-136/2014). It was conducted in accordance with the principles of Good Clinical Practice and the Declaration of Helsinki. All patients provided their informed consent to participate in the study. Prior to enrollment, a detailed explanation of the study objectives, procedures, and potential risks and benefits was given to each patient. They were also informed about their right to withdraw from the study at any time without any negative consequences for their medical care. Patients were encouraged to ask questions and seek clarification before making their decision to participate in the study.

### 2.2. Study Participants

The study included 128 patients, different in terms of gender (56 women, 54 men) and age (from 18 to 76 years old). The examined patients underwent surgical treatment of lumbar discopathy at the Neurosurgery Department of the T. Marciniak Lower Silesian Specialist Hospital (Poland). Qualification for surgical treatment followed a protocol that included a consultation with a neurosurgeon in the outpatient clinic (for elective admissions) or a consultation with a neurosurgeon on duty (for emergency admissions). Patients were consecutively enrolled among all individuals undergoing first-time lumbar spine surgery for discogenic LBP at the participating center during the study period. Eligibility was determined strictly according to predefined inclusion and exclusion criteria, without selective inclusion by treating clinicians.

### 2.3. Qualification Procedure

Patients who underwent lumbar spine surgery for the first time were included in the study. All patients diagnosed with discopathy in the lumbar spine and meeting strictly defined inclusion criteria were asked to take part in the research. The inclusion criteria were as follows: 1. age 18 years and older, and the need for the first surgery; 2. confirmed diagnosis of lumbar disc disease; 3. presence of neurological symptoms including but not limited to lower limb paresis, monoplegia, sphincter dysfunction, sensory disturbances, pain, and sexual dysfunction; 4. written informed consent to participate in the study; 5. patient attendance in the second stage of the study, i.e., volunteering to participate in the research. The exclusion criteria included the following: 1. refusal to participate in the study, 2. age lower than 18 years old, 3. presence of a mental disorder that prevented participation in the research, 4. failure of the participant to report for the second stage of the study, 5. other disorders that prevented the completion of the questionnaire, such as cognitive disorders. Ultimately, 110 patients were qualified to participate in the research and completed the study protocol.

### 2.4. Study Course

The decisions for qualifying patients for surgery were based on the assessment of the clinical condition of the patient and the results of radiological tests, such as magnetic resonance imaging (MRI) or computed tomography (CT). Indications encompassed factors such as the severity of symptoms, presence of neurological deficits, and evidence of structural abnormalities in the lumbar spine, as identified through imaging. The decision for surgical intervention aimed at addressing cases where conservative treatment failed or in the presence of complications, such as spinal root or cord compression. Surgical treatment followed by structured postoperative neurorehabilitation was selected in patients with chronic discogenic LBP who had persistent symptoms despite prior conservative management or presented with clinically significant neurological deficits, such as motor weakness, sensory disturbances, or progressive pain. All patients enrolled in the study underwent a comprehensive evaluation and received conservative therapy, incorporating physical therapy, before being deemed eligible for surgical intervention. The decision to proceed with surgery was contingent on a meticulous assessment of individual responses to conservative treatments, the persistence of symptoms, and the overall clinical presentation. This strategy was implemented to reserve surgical intervention for cases where conservative measures failed to produce satisfactory outcomes or when specific indications for surgical intervention were identified.

### 2.5. Neurorehabilitation Procedures

Following neurosurgery, all patients participated in a structured neurorehabilitation program designed to complement the surgical intervention and implemented as part of standard postoperative care at our center. The rehabilitation program commenced immediately after surgical stabilization and medical clearance, under the supervision of a multidisciplinary team comprising physiatrists, neurologists, physiotherapists, and rehabilitation nurses. During hospitalization, patients typically engaged in supervised neurorehabilitation sessions once daily, with each session lasting approximately 30–45 min. After hospital discharge, rehabilitation was continued on an outpatient basis, usually 2–3 times per week, for a minimum period of three months postoperatively. The exact frequency and duration of outpatient sessions were adjusted according to individual clinical status, neurological deficits, pain levels, and functional capacity.

The rehabilitation program consisted of individualized physical therapy sessions tailored to each patient’s specific neurological and functional impairments. Treatment strategies included passive and active range-of-motion exercises, muscle strengthening, proprioceptive training, balance enhancement, gait training, and manual therapy aimed at restoring spinal mobility and alignment. Progression of rehabilitation intensity and complexity was guided by clinical assessment, patient tolerance, neurological recovery, and functional performance rather than by rigid, time-based criteria. Pain management techniques, including TENS and therapeutic massage, were incorporated as adjunctive interventions to optimize patient comfort, facilitate participation in active rehabilitation, and support adherence to therapy. In addition to supervised sessions, patients received detailed instructions for individualized home-based exercise programs and ergonomic recommendations intended to maintain mobility, improve strength, and support long-term recovery while reducing the risk of symptom recurrence.

Compliance with the neurorehabilitation program was monitored throughout the postoperative period by the treating neurologists and rehabilitation staff. Patients were regularly reassessed, and rehabilitation strategies were modified as needed to reflect evolving clinical conditions and functional capabilities. Overall, the neurorehabilitation intervention reflected a standardized framework of usual care at our center, with content and intensity individualized rather than delivered as a rigid, uniform protocol.

### 2.6. Outcome Measures

Upon admission to the department, qualified patients were asked to complete a questionnaire on sociodemographic and clinical data. All patients underwent neurological examination and assessment of neurological symptoms related to discopathy. The assessment was performed by a specialist neurosurgeon before the surgery and 3 months after the patient was discharged from the unit. The neurological examination included knee and ankle reflexes, surface and deep sensation testing, the Lasègue’s test for lumbar radiculopathy [degrees], and the toe-to-floor test [cm]. The presence of neurological symptoms that could accompany discopathy, i.e., lower limb paresis, monoplegia, sphincter dysfunction, sensory disturbances, pain, and sexual dysfunction, was also determined. Sexual dysfunction was assessed as part of the comprehensive neurological and clinical evaluation conducted by a specialist neurosurgeon before surgery and three months after hospital discharge. The assessment was based on a structured clinical interview focusing on patient-reported sexual difficulties potentially related to lumbar disc pathology and neurological impairment. This variable was pre-specified in the study protocol as a clinically relevant symptom; however, no validated sexual function questionnaire was used. Additionally, the following were analyzed along with the assessment of the severity of pain: muscle atrophy of the lower limbs, the presence of scoliosis, the presence of lumbar pain, freedom of lower limb positioning, limitations in active or passive mobility of the thigh, lower leg, foot, and lumbar section. The results of the tests of knee and ankle reflexes of the lower limbs were analyzed, as well as detailed results of sensory disorders tests.

### 2.7. Statistical Analysis

Statistical analysis was performed using Statistica 12 software (StatSoft, Inc., Tulsa, OK, USA). Arithmetic means, medians, standard deviations, and range of variation (extreme values) were calculated for quantitative variables. For qualitative variables, their frequency of occurrence (percentages) was calculated. All examined quantitative variables were tested using the Shapiro–Wilk test to determine the type of distribution. The comparison of the results before and 3 months after surgery was performed using the parametric Student’s *t*-test for dependent samples or the non-parametric Wilcoxon test. The comparison of qualitative variables before and 3 months after the surgery was performed using the chi-squared test (χ^2^). Additionally, the relationship between the selected variables was determined using the Pearson correlation test. Statistically significant results were accepted at a significance level of *p* < 0.05.

## 3. Results

### 3.1. Patients’ Characteristics

Ultimately, 110 patients participated in the study, the detailed characteristics of whom are presented in Table 1.

### 3.2. Patients’ Disease Perception

Important data regarding the characteristics of the main disease before the surgery are included in Table 2. It was noticed that more than 50% of participants related their health issues to work (59.09%, *n* = 65), the remainder to sports, household duties, or other factors. Moreover, 67.27% (*n* = 74) of the study participants declared that there was no family history of disc disease. In all patients, the disc disease involved the lumbar region of the spine and L3-L4-L5 level. The majority of the patients’ health issues lasted between 5 and 10 years (41.82%, *n* = 46). More than 95% of the research participants reported that they experienced pain associated with discopathy, and in most cases this was chronic (85.45%, *n* = 94).

### 3.3. Neurological Condition

Among the assessed neurological symptoms, statistically significant differences were observed in the case of sensory disorders (*p* < 0.0001), pain (*p* < 0.0001), and sexual dysfunction (*p* < 0.0001), as shown in Table 3. Sensory disorders occurred in 35 patients (31.82%) before the procedure, while the number of patients with sensory disorders decreased 3 months after the surgery and was 9 (8.18%). Pain was reported by 94 patients (85.45%) before the procedure; only approximately 8 respondents (7.27%) reported pain after the surgery. The opposite was observed in the case of sexual dysfunction. Before the procedure, such problems were the case of approximately 2 people (1.82%), while the percentage was much higher after the surgery and affected 49 study participants (44.55%).

Based on the neurological examination, differences were observed in the assessment of the presence of scoliosis, as shown in Table 4. Three months after the procedure, no scoliosis was noticed in the study participants. Before the procedure, such symptoms were observed in approximately 8 people (7.27%) (*p* = 0.0040). Moreover, statistically significant differences were observed in the occurrence of pain in the lumbar spine area (*p* < 0.0001). Before the procedure, that kind of pain was noticed in 82 patients (74.23%). Only 49 people (44.55%) reported pain three months after the procedure.

### 3.4. Movements and Pain

A similar situation was observed in the study of the limitation of active and passive mobility in the thigh area, as shown in Table 5. The surgical procedure did not statistically significantly improve or worsen that mobility.

The results of the assessment of active and passive lower leg mobility and the occurrence of pain during lower leg movement are presented in Table 6. It was observed that pain was present in 31 patients (28.18%) before the surgery; only 16 patients (14.55%) were affected by lower leg pain (*p* = 0.0136) 3 months after the surgery.

The results of the assessment of active and passive foot mobility and the occurrence of pain during foot movement are presented in Table 7. It was observed that pain was present in 30 patients (27.27%) before the surgery; the number of patients with foot pain decreased 3 months after the surgery, and the pain was the case of 15 patients (13.64%) (*p* = 0.0122).

The results of the assessment of active and passive mobility in the lumbar spine area and the occurrence of pain during movement in that part of the body are presented in Table 8. It was observed that limited active and passive mobility in the lumbar spine area and pain occurred in most people before the procedure. All results were statistically significant (*p* < 0.0001).

### 3.5. Deep Tendon Reflexes

A comparison of knee and ankle reflexes before and 3 months after the procedure is presented in Table 9. Statistically significant differences in the assessment of such reflexes were observed before and 3 months after the procedure (*p* < 0.0001). Before the procedure, a negative test result during the assessment of the knee reflex of the right lower limb was observed in almost 10% of people (9.09%); after the procedure that percentage was 0%. With regard to the left limb knee reflex, that percentage decreased—it was 7.27% before the procedure and 3.64% after the surgery. A decrease in the percentage of people with a negative result (no Achilles tendon reflex) was also observed in the assessment of the left lower limb ankle reflex (from approximately 13.64% to 10.91%), while in the case of the right lower limb ankle reflex, an increase was observed from approximately 8.18% to 14.55% 3 months after the surgery.

### 3.6. Superficial and Deep Sensory

The results of the assessment of superficial sensation, deep sensation, numbness, and tingling are shown in Table 10. No significant changes were observed in the case of the above variables. The results of the percentage distribution of people with given symptoms before and after 3 months after the procedure are similar. The procedure was not a factor that significantly reduced or intensified the occurrence of those symptoms.

### 3.7. Functional Tests

The results of functional tests before and 3 months after the procedure were also compared, which is summarized in Table 11. A statistically significant difference was observed in the results of the toe-to-floor test before and 3 months after the procedure (*p* < 0.0001). The average result before the procedure was 29.5 cm, and the average increased after the procedure and amounted to 42.8 cm, i.e., the range of motion improved and pain in the lumbar spine decreased. The Lasègue’s test results were found to be statistically insignificant.

## 4. Discussion

The fact is that most patients with IDD are treated conservatively, especially in the early stages of the condition, and there are several reasons for that. First, conservative therapy is often a safe and effective treatment option for IDD, especially when symptoms are not advanced yet. Conservative treatment may include such methods as physiotherapy, manual therapy, rehabilitation exercises, and pharmacotherapy to help reduce pain, improve spinal function, and increase overall patient comfort. Second, surgical interventions related to the treatment of IDD are usually reserved for cases in which conservative therapy has failed to produce the desired results or when serious complications occur, such as pressure on spinal roots or the spinal cord. Surgery can involve risks and a longer recovery period; therefore, it is avoided if possible. Third, conservative treatment can help patients manage chronic LBP; it is mainly about educating the patients on appropriate attitudes and habits that support a healthy spine and counteract deterioration as part of secondary prevention. The purpose of that approach is not only the treatment of the symptoms but also the prevention of future recurrences [29,30].

If conservative treatment fails and the patient’s condition worsens, surgical options are considered. The issue of performing the procedure in the case of chronic pain is still controversial. The multitude of surgical methods, different patient selection criteria, and inconsistent LBP assessment criteria make a definitive evaluation of that problem difficult [31]. Some studies, including randomized trials and literature reviews, indicate complications and deterioration of patients’ health in the post-surgery period [32].

Over the years, various methods have been used to evaluate the results of surgical treatment of intervertebral disc herniations. Researchers still use clinical criteria that take the subjective assessment into account, i.e., the level of pain relief, pain in the lumbosacral spine, and pain radiating along the lower limb in the form of sciatica. The level of mobility, the quality of the functional status after the surgery, and the persistence of neurological disorders are also considered [33,34].

It is reasonable to further verify the effectiveness of IDD surgery bearing in mind the clinical assessment and neurological symptoms, which directly translate into the patient’s sensation and satisfaction after the surgery, as well as the perception of their quality of life. Therefore, in this study, the presence of neurological symptoms, assessed before the surgery and 3 months after the procedure, was comprehensively analyzed. The main focus was on pain and sensory disturbances, as well as other symptoms that could suggest severe spinal cord compression, such as lower limb paresis, monoplegia, sexual dysfunction, and sphincter dysfunction.

Significant differences were found for sensory disturbances (32% of patients before the surgery, 8% after the procedure, *p* = 0.0001), the occurrence of pain (86% before the surgery, 7% after the procedure, *p* < 0.0001), and sexual dysfunction (2% before the surgery, 45% after the procedure, *p* < 0.0001). The relief of pain after the surgery is evident and does not require further comment, especially in the light of previous results; a reduction in the incidence of sensory disturbances has also been reported by other authors [35], while some have observed an increase in their incidence [36,37]. However, a significantly increased incidence of sexual dysfunction may cause anxiety in patients. Its analysis would require more detailed information on the causes of the disorders, their severity, and the nature of the changes.

The observed increase in reported sexual dysfunction at the postoperative follow-up should be interpreted with caution. Several factors may contribute to this finding. First, sexual dysfunction was assessed using a structured clinical interview rather than a validated questionnaire, which may increase sensitivity to subjective complaints and reporting variability. Second, postoperative follow-up visits may provide patients with greater opportunity and willingness to report intimate symptoms that were underreported or not prioritized before surgery due to pain or neurological impairment. Third, early postoperative factors such as residual pain, fear of movement, psychological stress, altered body image, or temporary activity restrictions may negatively influence sexual function, despite overall neurological improvement.

A large-scale study by Fritzel et al. [38] did not reveal any significant sexual complications; however such problems have already been widely reported in the literature [39,40,41]. Mannion et al. [37] provide detailed data based on the analysis of 2282 patients within one year after the lumbar spine surgery due to degenerative changes. The researchers note that 687 (30.1%) patients reported complications, the largest percentage of which were sensory disorders (36% of people with complications) or pain (26%), followed by motor problems (8%), neurological disorders (11%), and wound healing problems (6%).

Apart from the above issues, other neurological symptoms were also analyzed in the patients under study. In particular, passive and active mobility of the lower limbs, lumbar spine, muscle tone, and strength of the lower limbs, as well as knee and ankle reflexes were assessed. A significant difference was observed in terms of the incidence of lumbar spine scoliosis in the patients. Before the surgery, scoliosis was noted in eight patients (7.27%), whereas 3 months after surgery it was found in none of the patients (*p* = 0.0040). This is crucial in terms of improving patients’ quality of life and may indicate the success of the surgery in restoring function and eliminating pain in the lumbar spine.

In turn, the surgery did not significantly affect muscle atrophy and free positioning of the lower limbs, nor did it limit the active and passive mobility of the thigh, lower leg, and foot; however, the frequency of pain when moving the lower leg (*p* = 0.0136) and foot decreased (*p* = 0.0122). After the procedure, active and passive mobility of the spine increased (*p* < 0.001), and the frequency of pain during movement of the lumbar spine decreased from 51.82% to 23.64% (*p* < 0.0001).

The analysis of the ankle and knee reflexes revealed that there was a significant reduction in the frequency of negative reflexes 3 months after surgery (*p* < 0.0001). The results suggest that surgical treatment of lumbar discopathy significantly improves knee reflexes in the lower limbs, especially in the right limb. Nevertheless, there are still some limitations in the normalization of ankle and knee reflexes, which may require further analysis and/or more intensive rehabilitation.

The assessment of superficial sensation, deep sensation, numbness, and tingling did not reveal statistically significant differences between the pre-surgery examination and the one performed 3 months after the procedure. The results for the assessment of superficial sensation, deep sensation, numbness, and tingling before and after the surgery show no significant changes in those variables. This means that the surgery did not have a significant impact on those specific neurological aspects in the patients under study. It is important to understand that surgery does not always result in an improvement in every aspect of neurological function, and results may vary depending on individual patients.

Moreover, the Lasègue’s test showed no significant differences, while a major improvement was noticed when performing the toe-foot test. The mean score before the surgery was 29.5 cm, while it was 42.8 cm (*p* < 0.0001) after the procedure. The obtained results suggest that the surgery had a positive impact on improving the range of motion and reducing lumbar spine pain, which was confirmed by the results of the toe-to-floor test. However, in the case of the Lasègue’s test, the results remained unchanged. This emphasizes that surgery can have different effects depending on patients’ specific motor function or symptoms.

Interesting results are presented by Radziszewski [42], who assessed—by way of a neurological examination (Lasègue’s test, patellar and ankle reflexes, superficial sensation and vibration, sphincter function and muscle strength)—665 patients with lumbar discopathy L4-L5 and L5-S1 who underwent conservative treatment or surgery. The results showed that the conservatively treated group (*n* = 348) did not show significant progression of neurological symptoms over the period of five years. However, the group of patients who underwent the surgery (*n* = 317) observed a systematic worsening of neurological symptoms in the second year after the surgery and later on. Patients at the age of 50 or older observed higher levels of neurological disorders than younger and middle-aged ones. In the period immediately after the surgery, the improvement rate of neurological disorders was higher than in the corresponding period of conservative therapy. However, in long-term studies, the average number of neurological symptoms turned out to be higher in the group of surgically treated patients. In another study, the same author [43] concluded that the functional status of patients with basic lumbar discopathy was determined by the neurological condition and the intensity of pain, and surgical treatment brought a significant improvement in the functional status of the patients, which could deteriorate in subsequent years of treatment.

The narrative review by Nv et al. [18] deals with pathogenesis, the most relevant factors associated with the occurrence of neurological disorders and deficits in patients with lumbar discopathy, as well as the factors with prognostic significance for the recovery process. It turns out that most studies of prognostic factors for the improvement of movement disorders prove that the muscle strength parameter is an important prognostic factor of the functional state. The timing of the surgery is also an important prognostic factor, as patients operated on more quickly (35 days) after the onset of paresis recovered fully, in contrast to those who underwent the surgery at a later stage (69 days). There are also controversial studies reporting that surgical treatment does not give a better prognosis for the improvement of motor deficits compared to conservative treatment.

The fact is that there is a need to implement a personalized approach in the treatment process of patients, which includes a thorough neurological examination and assessment of neurological deficits or functional impairments to tailor therapy to the individual needs and characteristics of each patient. Only such an approach can result in optimal therapeutic outcomes and increase the chance of improving the quality of life of patients.

It should also be noted that current clinical guidelines for chronic LBP emphasize structured conservative multimodal interventions [44,45], such as exercise-based rehabilitation combined with patient education (e.g., Back School or spine school programs), as first-line treatment options [46,47]. These approaches aim to improve pain coping strategies, functional capacity, and self-management, and are effective for a substantial proportion of patients without significant neurological compromise. However, a subset of patients with discogenic LBP experience persistent symptoms despite adequately applied conservative care or present with clinically relevant neurological deficits, including motor weakness, sensory disturbances, or progressive functional limitation. In such cases, surgical intervention followed by structured postoperative neurorehabilitation may be considered as a subsequent therapeutic pathway. The present study reflects outcomes in this specific patient population, in whom surgery was selected based on clinical indications and failure of prior conservative treatment, rather than as an alternative to first-line multimodal conservative programs [20].

Our findings suggest that, in appropriately selected patients, the combination of surgical treatment and postoperative neurorehabilitation can lead to meaningful early improvements in neurological status and functional mobility. Importantly, these results should not be interpreted as diminishing the role of conservative multimodal care, but rather as supporting a stepwise, patient-centered treatment strategy in which surgical and rehabilitative interventions are reserved for cases where conservative approaches are insufficient or neurological deterioration is evident.

### 4.1. Strengths and Limitations

The present study has several notable strengths. It addresses a clinically important and topical issue by providing a comprehensive evaluation of neurological and functional outcomes in patients undergoing lumbar disc surgery. The inclusion of patients from both elective and emergency admissions enhances the clinical representativeness of the cohort and reflects real-world neurosurgical practice. The relatively large sample size strengthens the robustness of the analyses and allows for a detailed assessment of multiple neurological domains. Furthermore, the use of standardized self-reported and clinical assessments, applied consistently across the study population, supports the reliability and comparability of the collected data.

There are also potential methodological limitations to this study. First, the absence of a control group limits the ability to isolate the specific effects of postoperative neurorehabilitation from those of surgical intervention itself, and the observed improvements should therefore be interpreted as the combined effect of surgery and subsequent rehabilitation. Second, the study was conducted at a single medical center, with all patients undergoing surgery at one institution, which may reduce the generalizability of the findings to other healthcare settings. In addition, the study was conducted in one region of Wroclaw (Poland), and the results may not be fully applicable to regions with different healthcare systems, rehabilitation resources, or patient populations. Another limitation of this study is the relatively short follow-up period of three months after surgery. While this timeframe allows for the assessment of early postoperative neurological and functional outcomes, it may not fully capture long-term recovery trajectories, delayed complications, or the durability of treatment effects. Sexual dysfunction was evaluated using a structured clinical interview rather than a validated, disease-specific questionnaire, which may limit the precision and comparability of this outcome. Furthermore, no a priori sample size calculation or formal power analysis was performed, as the sample size was determined pragmatically based on patient availability, which may limit the ability to detect smaller effect sizes for certain outcomes. Nevertheless, the study provides clinically relevant real-world data on early neurological and functional outcomes following lumbar spine surgery combined with postoperative neurorehabilitation.

Future studies should complement self-reported assessments with objective clinical and functional measures to provide a more comprehensive evaluation of postoperative recovery. The use of validated outcome instruments, quantitative functional testing, and standardized neurological scoring systems may help improve comparability across studies. Longer follow-up periods are also needed to assess the durability of neurological and functional improvements and to capture delayed or transient effects of treatment. Additionally, future research should explore the influence of patient-related factors, such as comorbidities and baseline functional status, on recovery trajectories.

### 4.2. Clinical Implications

The findings of this study have several clinically relevant implications. First, the observed early improvement in neurological status and functional outcomes supports the role of neurosurgical intervention followed by structured postoperative neurorehabilitation in appropriately selected patients with chronic discogenic LPB and neurological deficits. Second, the multidimensional assessment of neurological symptoms highlights the importance of comprehensive postoperative evaluation, as recovery may vary across motor, sensory, and functional domains. From a clinical perspective, the results underscore the value of integrating neurorehabilitation early after surgery to support neurological recovery, optimize functional mobility, and facilitate return to daily activities. The variability observed across individual symptoms also emphasizes the need for personalized rehabilitation strategies and close postoperative monitoring.

## 5. Conclusions

Lumbar spine surgery can be effective in reducing pain and improving motor function in patients with IDD. The integration of lumbar spine surgery with structured neurorehabilitation significantly improves neurological and functional outcomes in patients with discogenic LBP. This combined approach notably enhances spinal mobility, reduces pain, restores deep reflexes, and improves lower limb mobility. The findings support the adoption of comprehensive postoperative neurorehabilitation protocols to optimize patient recovery and highlight the importance of individualized rehabilitation programs tailored to specific patient needs. Future research should focus on long-term follow-up studies to evaluate sustained improvements and refine rehabilitation strategies further.

## Figures and Tables

**Table 1:** Characteristics of the patients who took part in the study.

Characteristics of the Study Group (*n* = 110)		
Age [years]	$\bar{x}$	44.8
	Min–Max	18.0–76.0
	SD	13.1
Body height [cm]	$\bar{x}$	170.6
	Min–Max	152.0–193.0
	SD	8.8
Body weight [kg]	$\bar{x}$	76.6
	Min–Max	44.0–120.0
	SD	16.0
BMI [kg/m^2^]	$\bar{x}$	26.2
	Min–Max	17.6–39.1
	SD	4.5
Gender	female	50.91%
		*n* = 56
	male	49.09%
		*n* = 54

Abbreviations: *n*, count; $\bar{x}$, mean; Min, minimum value; Max, maximum value; SD, standard deviation; cm, centimeters; kg, kilograms; m^2^, square meters.

**Table 2:** Characteristics of the main disease before the neurosurgery.

Characteristics of the Studied Disease (*n* = 110)		
Which section of your spine is affected by the disc disease?	the lumbar spine	100.00% *n* = 110
What do you associate your health issues with?	work	59.09% *n* = 65
	sport	10.00% *n* = 11
	housework	17.27% *n* = 19
	other	13.64% *n* = 15
Is there a history of lumbar disc disease in your family?	no	67.27% *n* = 74
	yes	32.73% *n* = 36
How many years have you had lumbar disc disease?	1–5 years	24.55% *n* = 27
	5–10 years	41.82% *n* = 46
	10–15 years	8.18% *n* = 9
	>15 years	25.45% *n* = 28
How do you rest after work?	sitting	32.73% *n* = 36
	lying	53.64% *n* = 59
	staying physically active	13.64% *n* = 15
Do you experience pain related to your lumbar disc?	no	3.64% *n* = 4
	yes	96.36% *n* = 106
What is the nature of the pain?	chronic	85.45% *n* = 94
	acute (short-term, e.g., physical effort-related)	14.55% *n* = 16

Abbreviations: *n*, count; %, percentage.

**Table 3:** Comparison of neurological functions before and 3 months after the neurosurgery.

Variables		Before the Surgery	After the Surgery	*p*-Value *
Lower limb paresis	No (*n*)	62	49	*p* = 0.2151
	%column	56.36%	44.55%	
	Yes (*n*)	48	61	
	%column	43.64%	55.45%	
Monoplegia	No (*n*)	109	110	*p* = 0.6052
	%column	99.09%	100.00%	
	Yes (*n*)	1	0	
	%column	0.91%	0.00%	
Sphincter complications	No (*n*)	103	98	*p* = 0.4867
	%column	93.64%	89.09%	
	Yes (*n*)	7	12	
	%column	6.36%	10.91%	
Sensory disturbances	No (*n*)	75	101	*p* < 0.0001
	%column	68.18%	91.82%	
	Yes (*n*)	35	9	
	%column	31.82%	8.18%	
Pain	No (*n*)	16	102	*p* < 0.0001
	%column	14.55%	92.73%	
	Yes (*n*)	94	8	
	%column	85.45%	7.27%	
Sexual dysfunction	No (*n*)	108	61	*p* < 0.0001
	%column	98.18%	55.45%	
	Yes (*n*)	2	49	
	%column	1.82%	44.55%	

Notes: * test χ^2^. Abbreviations: *n*, count; %, percentage.

**Table 4:** Comparison of neurological examination results related to muscle atrophy and lower limb free positioning before and 3 months after neurosurgery.

Variable		Before the Surgery	After the Surgery	*p*-Value *
Muscle atrophy of the lower limbs	No (*n*)	108	107	*p* = 0.6510
	%column	98.18%	97.27%	
	Yes (*n*)	2	3	
	%column	1.82%	2.73%	
Scoliosis	No (*n*)	102	110	*p* = 0.0040
	%column	92.73%	100.00%	
	Yes (*n*)	8	0	
	%column	7.27%	0.00%	
Low back pain	No (*n*)	28	61	*p* < 0.0001
	%column	25.77%	55.45%	
	Yes (*n*)	82	49	
	%column	74.23%	44.55%	
Free positioning of the right lower limb	No (*n*)	3	10	*p* = 0.0682
	%column	2.73%	9.09%	
	Yes (*n*)	107	100	
	%column	97.27%	90.91%	
Free positioning of the left lower limb	No (*n*)	4	10	*p* = 0.1011
	%column	3.67%	9.09%	
	Yes (*n*)	105	100	
	%column	96.33%	90.91%	

Notes: * test χ^2^. Abbreviations: *n*, count; %, percentage.

**Table 5:** Comparison of the results of the assessment of active and passive mobility limitation and pain in the thigh area before and 3 months after the neurosurgery.

Variable		Before the Surgery	After the Surgery	*p*-Value *
Passive mobility limitation in the thigh area	No (*n*)	101	100	*p* = 0.8103
	%column	91.82%	90.91%	
	Yes (*n*)	9	10	
	%column	8.18%	9.09%	
Active mobility limitation in the thigh area	No (*n*)	104	99	*p* = 0.2068
	%column	94.55%	90.00%	
	Yes (*n*)	6	11	
	%column	5.45%	10.00%	
Pain when moving the thigh	No (*n*)	80	79	*p* = 0.8803
	%column	72.73%	71.82%	
	Yes (*n*)	30	31	
	%column	27.27%	28.18%	

Notes: * test χ^2^. Abbreviations: *n*, count; %, percentage.

**Table 6:** Comparison of the results of the assessment of active and passive mobility limitation and pain in the lower leg before and 3 months after the neurosurgery.

Variable		Before the Surgery	After the Surgery	*p*-Value *
Passive mobility limitation in the lower leg area	No (*n*)	100	100	*p* = 1.0000
	%column	90.91%	90.91%	
	Yes (*n*)	10	10	
	%column	9.09%	9.09%	
Active mobility limitation in the lower leg area	No (*n*)	103	99	*p* = 0.3252
	%column	93.64%	90.00%	
	Yes (*n*)	7	11	
	%column	6.36%	10.00%	
Pain when moving the lower leg	No (*n*)	79	94	*p* = 0.0136
	%column	71.82%	85.45%	
	Yes (*n*)	31	16	
	%column	28.18%	14.55%	

Notes: * test χ^2^. Abbreviations: *n*, count; %, percentage.

**Table 7:** Comparison of the results of the assessment of active and passive mobility limitation and pain in the foot area before and 3 months after the neurosurgery.

Variable		Before the Surgery	After the Surgery	*p*-Value *
Passive mobility limitation in the foot area	No (*n*)	98	100	*p* = 0.6351
	%column	89.09%	90.91%	
	Yes (*n*)	12	10	
	%column	10.91%	9.09%	
Active mobility limitation in the foot area	No (*n*)	102	98	*p* = 0.3482
	%column	92.73%	89.09%	
	Yes (*n*)	8	12	
	%column	7.27%	10.91%	
Pain when moving the foot	No (*n*)	80	95	*p* = 0.0122
	%column	72.73%	86.36%	
	Yes (*n*)	30	15	
	%column	27.27%	13.64%	

Notes: * test χ^2^. Abbreviations: *n*, count; %, percentage.

**Table 8:** Comparison of the results of the assessment of active and passive mobility limitation and pain in the lumbar spine area before and 3 months after the neurosurgery.

Variable		Before the Surgery	After the Surgery	*p*-Value *
Passive mobility limitation in the lumbar spine area	No (*n*)	69	100	*p* < 0.0001
	%column	62.73%	90.91%	
	Yes (*n*)	41	10	
	%column	37.27%	9.09%	
Active mobility limitation in the lumbar spine area	No (*n*)	69	96	*p* < 0.0001
	%column	62.73%	87.27%	
	Yes (*n*)	41	14	
	%column	37.27%	12.73%	
Pain with movement of the lumbar spine	No (*n*)	53	84	*p* < 0.0001
	%column	48.18%	76.36%	
	Yes (*n*)	57	26	
	%column	51.82%	23.64%	

Notes: * test χ^2^. Abbreviations: *n*, count; %, percentage.

**Table 9:** Comparison of knee and ankle reflex assessment results before and 3 months after the neurosurgery.

Variable		Before the Surgery	After the Surgery	*p*-Value *
Knee-jerk reflex of the right limb	− (*n*)	10	0	*p* < 0.0001
	%column	9.09%	0.00%	
	+ (*n*)	47	26	
	%column	42.73%	23.64%	
	++ (*n*)	39	84	
	%column	35.45%	76.36%	
	+++ (*n*)	14	0	
	%column	12.73%	0.00%	
Knee-jerk reflex of the left limb	− (*n*)	8	4	*p* < 0.0001
	%column	7.27%	3.64%	
	+ (*n*)	47	17	
	%column	42.73%	15.45%	
	++ (*n*)	43	82	
	%column	39.09%	74.55%	
	+++ (*n*)	12	7	
	%column	10.91%	6.36%	
Ankle-jerk reflex of the right limb	− (*n*)	9	16	*p* < 0.0001
	%column	8.18%	14.55%	
	+ (*n*)	58	29	
	%column	52.73%	26.36%	
	++ (*n*)	32	62	
	%column	29.09%	56.36%	
	+++ (*n*)	11	3	
	%column	10.00%	2.73%	
Ankle-jerk reflex of the left limb	− (*n*)	15	12	*p* < 0.0001
	%column	13.64%	10.91%	
	+ (*n*)	63	23	
	%column	57.27%	20.91%	
	++ (*n*)	21	71	
	%column	19.09%	64.55%	
	+++ (*n*)	11	4	
	%column	10.00%	3.64%	

Notes: * test χ^2^. Abbreviations: *n*, count; %, percentage. Reflex grades: −: absent; +: hypoactive; ++: normal; +++: hyperactive.

**Table 10:** Comparison of the results of the assessment of superficial sensation, deep sensation, numbness, and tingling before and 3 months after the neurosurgery.

Variable		Before the Surgery	After the Surgery	*p*-Value *
Superficial sensation	Weaker (*n*)	71	70	*p* = 0.5287
	%column	64.55%	63.64%	
	Proper (*n*)	35	35	
	%column	31.28%	31.82%	
	Hyperesthesia (*n*)	4	5	
	%column	3.64%	4.55%	
Deep sensation	Weaker (*n*)	3	7	*p* = 0.1795
	%column	2.73%	6.36%	
	Proper (*n*)	107	102	
	%column	97.27%	92.73%	
	Hyperesthesia (*n*)	0	1	
	%column	0.00%	0.91%	
Numbness	No (*n*)	88	92	*p* = 0.5746
	%column	80.00%	83.64%	
	Yes (*n*)	22	18	
	%column	20.00%	16.36%	
Tingling	No (*n*)	93	94	*p* = 0.8279
	%column	84.55%	85.45%	
	Yes (*n*)	17	16	
	%column	15.45%	14.55%	

Notes: * test χ^2^. Abbreviations: *n*, count; %, percentage.

**Table 11:** Comparison of the results of the toe-to-floor test and Lasègue’s test before and 3 months after the neurosurgery.

Variables		*n*	$\bar{x}$	Me	Min	Max	SD	*p*-Value
Toe-to-floor test [cm]	Before the surgery	110	29.5	30.0	10.0	70.0	8.9	*p* < 0.0001 **
	After the surgery	110	42.8	45.0	5.0	110.0	23.1	
Lasègue’s symptom—right side [degrees]	Before the surgery	110	58.0	50.0	10.0	90.0	23.5	*p* = 0.8983 *
	After the surgery	110	57.6	50.0	10.0	90.0	23.9	
Lasègue’s symptom—left side [degrees]	Before the surgery	110	61.1	60.0	10.0	90.0	21.0	*p* = 0.9491 *
	After the surgery	110	60.9	60.0	10.0	90.0	21.2	

Notes: * Student’s *t*-test; ** Wilcoxon test. Abbreviations: *n*, count; $\bar{x}$, mean; Me, median; Min, minimum value; Max, maximum value; SD, standard deviation; cm, centimeters.

## Data Availability

The raw data supporting the conclusions of this article will be made available by the authors on request.
